# The Hordaland Women's Cohort: A prospective cohort study of incontinence, other urinary tract symptoms and related health issues in middle-aged women

**DOI:** 10.1186/1471-2458-8-296

**Published:** 2008-08-23

**Authors:** David Jahanlu, Samera Azeem Qureshi, Steinar Hunskaar

**Affiliations:** 1 Section for General Practice, Department of Public Health and Primary Health Care, University of Bergen, Norway

## Abstract

**Background:**

Urinary incontinence (UI) is a prevalent symptom in middle-aged women, but data on incidence is limited and rarely reported. In order to analyze incidence, remission, or development patterns of severity and types of UI, we have established a 15-year prospective cohort (1997–2012).

**Methods:**

The Cohort is based on the national collection of health data gathered from county studies (CONOR). Hordaland Health Study (HUSK) is one of them from Hordaland County. Each of the county studies may have local sub-studies and our Cohort is one of them. The Cohort included women aged 40–45 in order to have a broad approach to women's health including UI and other lower urinary tract symptoms (LUTS). A onefifth random sampling from HUSK was used to create the Cohort in 1997–1999. For the necessary sample size a preliminary power calculation, based on a 70% response rate at inclusion and 5% annual attrition rates was used. The Cohort is planned to collect data through questionnaires every second year for the 15-year period from 1997–2012.

**Discussion:**

The Cohort represents a relatively large random sample (N = 2,230) of about 15% of the total population of women born between 1953–57 in the county of Hordaland. Our data shows that the cohort population is very similar to the source population. The baseline demographic, social and medical characteristics of the Cohort are compared with the rest of women in HUSK (N = 7,746) and there were no significant differences between them except for the level of education (P = 0.001) and yearly income (P = 0.018), which were higher in the Cohort population. Urological characteristics of participants from the Cohort (N = 1,920) were also compared with the other participants (N = 3,400). There were no significant statistical differences except for somewhat more urinary continence (P = 0.04), more stress incontinence (P = 0.048) and smaller amount of leakage (P = 0.015) in the Cohort. In conclusion, the Cohort ispopulation-based, with little selection bias, and thus is a rather unique study forinvestigating UI and LUTS in comparison with many other projects with similar purposes.

## Background

Urinary symptoms are an integral part of the transition from the premenopausal to the postmenopausal state. Decrease in natural hormones and other ageing processes in perimenopausal women induce changes in the urinary tract and vagina, and women become more susceptible to the development of bladder storage symptoms like urinary frequency, urgency and stress incontinence. Beside urinary incontinence (UI), pelvic organ prolapse may occur. UI is a prevalent problem in middle-aged women, but data on incidence are limited and rarely reported [1-3]. The prevalence of UI increases with age. A recent major study found a gradual increase in the prevalence of UI throughout adulthood until the fifth decade, it after which it stabilized and even registered slight decrease up to age 70; after that, it started to rise again [4].

Epidemiological and clinical studies conducted in various populations have revealed risk factors and contributing variables for UI and other lower urinary tract symptoms (LUTS). Factors such as smoking, menopause, restricted mobility, chronic cough, chronic straining due to constipation and uro-genital surgery have not been as rigorously studied as age, parity and obesity. Data on risk factors for the incidence of UI, remission and natural history of LUTS is limited and has been derived mainly from cross-sectional studies. This limits generalisation and restricts interpretation of causality. To show the temporal ordering between risk factors and onset of UI, a prospective or longitudinal design is necessary [2,3]. It has proven difficult to show predictors for incident UI except for pregnancy and delivery and some specific, but rare conditions like stroke and other neurological diseases. It has been documented that remission can take place, but its predictors are not well understood. It may be related to natural recovery or to medical care, but variation caused by unreliable measurements cannot be excluded [2].

Most previous studies on incidence have been small [5,6], have had few follow-ups or have a limited description of the type and severity of UI [5,7]. UI is part of a broader picture of urinary symptoms in middle-aged women. Both overactive bladder and other LUTS are prevalent [8], and several other health issues may be relevant and related in this age range of age. To answer some of the current issues we started a prospective cohort in 1997, *The Hordaland women's cohort*. This Cohort included women aged 40–45 years with a planned 15-year follow-up, to have a broad approach to women's health including UI and other LUTS. In 2007 the Cohort reached a 10-year follow up, and the present paper focuses on the design of the study, inclusion and recruitment, response rates and loss to follow-up, together with baseline characteristics of the cohort population.

## Methods

The Hordaland Women's Cohort is based on the publicly managed system for epidemiological research in Norway. The first level of this system consists of CONOR, the Cohort of Norway, which is a national collection of health data gathered from the second level i.e. the county studies. Each of the county studies may in turn have local sub-studies for specific purposes, diseases or populations and this is the third level.

### CONOR

Norway has particular advantages for epidemiological research because it is a survey-able and well-organized little country, where each individual has a unique ID number. This is the background for the foundation of CONOR [9]. CONOR is both the name of a collection of health data and blood samples, and of the collaboration between the Norwegian Institute of Public Health and the universities in Oslo, Bergen, Tromsø and Trondheim. In CONOR, regional data from 10 different epidemiological studies have been merged into a national database that is more representative of the Norwegian population than each of the individual sites. The first data was collected in 1994 and the last will be included in 2008. Altogether nearly 200000 people will be included.

The database consists of information obtained from questionnaires, a simple physical examination, analyses of blood samples, and frozen stored blood and/or DNA. The CONOR questions cover the following main topics: self-reported health and diseases such as diabetes, asthma, coronary heart disease, stroke and mental distress, musculoskeletal pains, family history of disease, risk factors and lifestyle, surrounding environment, social network and social support, education, work and housing, some occupations, use of medications and reproductive history (women). Most of the studies consist of a central core and several supplementary projects. The main purpose of CONOR is to study the aetiology of rare diseases by testing environmental, inheritable, cultural and social factors to describe the dispersion of diseases and risk factors by time, place and socio-demographic factors. One of the county health surveys was the Hordaland Health Study (HUSK) [10].

### HUSK

HUSK (1997–1999) was a joint epidemiological research project carried out by the National Health Screening Service in collaboration with the University of Bergen. The study population included all individuals in Hordaland County born between 1953–57 (29,335), and aged 40–44 at the time of the data collection.

A questionnaire was mailed to the invited people, with an invitation to a health check-up. The completed questionnaires were collected at the screening station, and each participant signed a written consent form. A total of 18,581 (8,598 men and 9,983 women) answered the questionnaire and came to clinical examinations, yielding a participation rate of 63% (57% for men and 70% for women). Baseline measurements included height, weight, waist and hip circumference, blood pressure, heart rate, non-fasting analyses of serum total cholesterol, HDL cholesterol, triglycerides, creatinine and glucose. Self-administered questionnaires including open-ended questions on occupation and industrial affiliation as well as use of medicinal and dietary supplements provided information on various health behaviours. One of the accessory questionnaires had a comprehensive section on LUTS. This questionnaire was randomly given to 65% of women at screening stations, to be filled in at home and sent back.

HUSK was then used as the basis for creating a prospective cohort of middle-aged women in Hordaland.

### The Hordaland Women's Cohort Study

#### Random sampling

The HUSK female population aged 40–45 was the source population for the Cohort. A random sampling of one-fifth of them was used. Those who came to the screening station received information about the Cohort. They eventually agreed to take part in the study by signing an informed consent form. For random sampling, the last digit of the personal ID number was used. For the necessary sample size a preliminary power calculation, based on a 70% response rate at inclusion and 5% annual attrition rates was used. With this calculation, at least 2,150 women should be asked to join at the baseline if at least 900 women should remain in the cohort after 10 years.

#### Questionnaire and variables

All information collected for HUSK participants formed the first data point (baseline dataset) for the Cohort members. Since then, postal questionnaires have been sent every second year to the Cohort members. The questionnaires have been almost identical each time and have four major parts. Table 1 shows the topics covered in the first 6 waves of the study.

**Table 1:** Topics covered in the questionnaires in the first six waves of the Cohort

Topics	1^st ^wave	2^nd ^wave	3^rd ^wave	4^th ^wave	5^th ^wave	6^th ^wave
Self-defined age				x	x	x
Self-rated health	x	x	x	x	x	x
Doctor visit last 6 months	x	x	x	x	x	x
Physical activity	x	x	x	x	x	x
Mammography		x		x		
Use of complementary medicine		x			x	x
Pelvic floor exercises	x	x	x	x	x	x
Smoking	x	x	x	x	x	x
Hysterectomy	x			x		
Oophorectomy	x			x		
Weight	x	x	x	x	x	x
Enuresis	x					
Regular menstruation	x	x	x	x	x	x
Last menstruation	x	x	x	x	x	x
Menopause symptoms	x	x	x	x	x	x
Intensity of menopause symptoms	x	x	x	x	x	x
Hormone therapy	x		x	x	x	x
Contraception	x	x	x	x	x	x
Who prescribed contraception				x	x	x
Dysuria	x	x	x	x	x	x
Urinary frequency	x	x	x	x	x	x
Nocturia	x	x	x	x	x	x
Urinary retention symptoms	x	x	x	x	x	x
Urgency		x	x	x	x	x
Stress UI	x	x	x	x	x	x
Urge UI	x	x	x	x	x	x
Other types of UI	x	x	x	x	x	x
UI frequency	x	x	x	x	x	x
UI amount	x	x	x	x	x	x
UI treatments	x	x	x	x	x	x
Bother of UI	x	x	x	x	x	x
Seeking medical help for UI	x	x	x	x	x	x
Drugs used yesterday	x	x	x	x	x	x
Name and reason for drug use	x	x	x	x	x	x

The first part (health, lifestyle and physical activity) covers variables like: age, self-rated general health, visits to doctor/hospital, physical activity, weight, pelvic floor exercises and smoking. This part also contains questions about complementary treatments such as acupuncture and homeopathy. When the questionnaire is returned, the International Classification of Primary Care (ICPC) [11] was used to code the reason for visiting a doctor or hospital.

The second part (contraception and menopause) asks about menstruation pattern and possible pre- and post-menopausal symptoms, the severity of the symptoms, use of hormones, contraception methods and a specific question about what kind of doctor (GP or specialist) prescribes contraceptives or intra-uterine devices (IUD).

The third part of the questionnaire (urinary conditions) contains two major sections. The first section is for all participants and contains nine questions about frequency of voiding each day, nocturia, bladder emptying, strength of urination, and experiences about leakage of urine in different situations. By combining the two questions about frequency and amount of leakage, the incontinence severity index (ISI, Sandvik index) [12] can be calculated. ISI is based on information about frequency (four levels) and amount of leakage (three levels). By multiplying these two, an index value (1–12) is determined. This index value is further categorized into a severity index with four levels of UI: slight (1–2), moderate (3–6), severe (8–9) and very severe (12) grade of UI. Typically slight incontinence stands for leakage of drops a few times a month, moderate incontinence daily leakage of drops, and severe incontinence larger amounts at least once a week. The severity index has been validated against a 48-hour "pad weighing" test among 303 incontinent women. According to this test, slight incontinence represented a mean leakage of 6 g/24 hours (95% CI 2–9), moderate incontinence means a leakage of 17 g/24 hours (95% CI 13–22) and severe incontinence a mean leakage of 56 g/24 hours (95% CI 44–67). The severity index is thus a semi-objective and quantitative measure, and does not include the woman's subjective perception of her leakage as being a problem or not. The ISI has been translated and validated by Spanish [13], Scottish [14] and American [15] groups who demonstrated that its reliability and responsiveness were also good. It has received the highest recommendation (grade A) from the 2^nd ^and 3^rd ^International Consultation on Incontinence [16].

The second section of the third part contains five questions only for women who have, or have had, urinary leakage. This section asks if there was any treatment or help for UI and also a question for self-rating how disturbing the urinary leakage is.

The last part of the questionnaire (consumption of drugs and complementary medicine) asks the participant if she used any kind of medicine the day before. A brief introduction explains the meaning of "medicine" as any drugs with or without prescription, in any form. If the answer is positive, then a chart is available to answer what was the medication, if it is a daily intake, and why she used it. The answer could be a diagnosis, name of a disease, a symptom or health effect. ATC (Anatomical Therapeutic Chemical Classification) [17] is used for coding the medicines and ICPC for coding the reason for using medication.

#### Recruitment, participants and representativeness

Among women born in 1953–1957 (N = 14,349) and invited to take part in HUSK, 9,983 (71.8%) came to a screening station. A total of 3,453 were selected by random sampling to participate in the Cohort, and 2,331 (67.5%) of them met. After oral and written information, 2,230 (95.7%) consented to take part in the study.

Baseline demographic, social, and medical characteristics of the Hordaland Women's Cohort (N = 2,230) compared with the source population in HUSK (N = 7,746) are shown in Table 2. The data shows that there are no significant differences between two groups except for education and annual family income, which is higher among women in the Cohort.

**Table 2:** Comparison of baseline socio-demographic characteristics between the Cohort and rest of the women in the Hordaland Health Study (HUSK).

	**HUSK minus the Cohort ** **(N = 7746)**		**The Cohort ** **(N = 2230)**		**P values**
	**N**	**%**	**N**	**%**	
	
**Age at inclusion (years)**					
40	1465	18.9	481	21.6	
41	1518	19.6	478	21.8	
42	1578	20.4	456	20.4	
43	1521	19.6	500	22.4	
44	1664	21.5	315	14.1	
**Marital status**					0.88
Not married	788	10.2	230	10.3	
Married	5800	74.9	1676	75.2	
Single	82	1.1	23	1.0	
Divorced	861	2.7	248	2.4	
Separated	212	2.7	53	2.4	
Registered partnerships	3	0.03	0	0	
**Education**					0.001
Elementary school	1616	20.7	408	18.2	
The lower secondary	2687	34.7	749	33.6	
The upper secondary	755	9.8	284	12.7	
University less than 4 years	1371	17.7	410	18.4	
University 4 years and more	1211	15.6	365	16.4	
Missing	106	1.4	14	0.6	
**Annual family income, NOK 1000**					0.018
0–199	1103	14.2	271	12.2	
200–399	2483	32.1	702	31.5	
> 400	2810	36.3	924	41.4	
Missing	1350	17.4	333	14.9	
**Parity**					0.768
0	544	7.0	166	7.4	
1	731	9.4	211	9.5	
2	2596	33.5	803	36.0	
3+	2802	36.1	794	35.6	
Missing	1073	13.9	265	11.9	
**Body mass index (kg/m2)**					0.328
Under-weight (< 18.5)	96	1.2	27	1.2	
Normal (18.5–24.9)	4582	59.2	1357	60.9	
Overweight (25–29.9)	2247	29.0	628	28.2	
Obesity	797	10.4	216	9.6	
Missing	24	0.3	2	0.1	
**Self-rated health**					0.170
Bad	91	1.2	18	0.8	
Not very good	1120	14.5	294	13.2	
Good	4791	61.7	1407	63.1	
Very good	1665	21.5	497	22.3	
Missing	79	1.0	14	0.6	
**Lifestyle and medical conditions**					0.199
Regular exercise	4519	58.4	1342	60.2	0.199
Daily smoking	2727	35.2	757	33.9	0.482
Asthma	503	6.5	155	7.0	0.444
Diabetes	87	1.1	23	1.0	0.715
Alcohol intake > 6 times/month	1417	18.3	418	18.8	0.095
Psychiatric problem/condition	1144	14.8	314	14.1	0.229

Table 3 compares urological characteristics between women who are in the Cohort with the rest of the women who answered the urological questionnaire. There were no significant statistical differences between them, except the fractions for urinary continence, distribution of type of incontinence and amount of leakage.

**Table 3:** Comparison of urological characteristics between the Cohort and the rest of the women in the Hordaland Health Study (HUSK)

	**HUSK minus the Cohort ** **(N = 3400)**		**The Cohort ** **(N = 1920)**		**P value**
	**N**	**%**	**N**	**%**	
	
**Dysuria episodes last 12 months**					0.763
No	2761	81.2	1541	80.3	
1–2 times	399	11.7	232	12.3	
3–5 times	102	3.0	61	3.2	
> 5 times	82	2.4	54	2.8	
Missing	56	1.6	32	1.7	
**Nocturia**					0.159
None	2445	71.9	1417	73.8	
1 time	767	22.6	405	21.1	
2 times	94	2.8	56	2.9	
> 2 times	43	1.3	14	0.7	
Missing	51	1.5	28	1.5	
**Feeling of incomplete emptying**	274	8.1	152	7.9	0.742
**Any urinary incontinence**	844	24.8	554	28.9	0.040
**Frequency of incontinence**					0.070
< 1/month	252	29.9	204	36.8	
> 1/month	305	36.1	187	33.8	
> 1/week	186	22.0	105	19.0	
Everyday	61	7.2	38	6.9	
Missing	40	4.7	20	3.6	
**Amount of leakage, distribution**					0.0149
Drops	509	60.3	360	65.0	
Small amounts	291	34.5	163	29.4	
Large amounts	15	1.8	10	1.8	
Missing	29	3.4	21	3.8	
**Incontinence Severity Index**					0.107
Slight (1–2)	447	53.0	325	61.4	
Moderate (3–6)	305	36.2	177	31.9	
Severe/very severe (8–9 & 12)	44	5.2	27	4.9	
Missing	48	5.7	25	2.0	
**Incontinence type distribution**					
Stress incontinence	437	51.8	307	55.4	
Urge incontinence	68	8.1	61	11.0	
Mixed incontinence	268	31.8	146	26.4	
Could not be classified	71	8.4	40	7.2	
**Duration of urinary incontinence**					0.875
0–5 years	536	63.5	352	63.5	
5–10 years	171	20.3	111	20.0	
> 10 years	86	10.2	52	9.4	
Missing	51	6.0	39	7.0	

### Ethics and formal approvals

HUSK and the Cohort were both approved by the Norwegian Data Inspectorate and Regional Committee for Medical Research Ethics. The cohort approval includes the right to obtain the full 11-digit personal identification numbers which make it possible to merge files and also extend the database with data from other sources, e.g. different national registers. All personnel and staff involved in the survey are bound by an oath of confidentiality.

## Discussion

We have been able to establish a cohort of more than 2,000 women for prospective studies of UI and related topics. The Cohort is planned to collect data every second year for the 15-year period between 1997–2012. The Cohort is population-based, with minimal selection bias. This Cohort is thus a rather unique study in comparison to many other projects with similar purposes.

The Cohort represents a relatively large random sample of about 15% of the total population of women born between 1953–57 in the county of Hordaland, which increases the generalization of our findings. The source study had a high participation rate, and of those who were invited to participate in the Cohort, almost all agreed to take part

The main objectives are to analyze incidence, remission, or development patterns in severity and types of UI. The variables and questions are adapted to current ICS definitions and the questionnaire contains many validated questions and indices from previous studies. The incontinence case definition is in accordance with the new definition of the ICS [18]. Furthermore, the UI data set is in accordance with the recommendations from the 3rd ICI [2], and several related health issues are covered to give a comprehensive data set for further analyses. At baseline, the Cohort had a prevalence of 28.9% for UI. The majority of women have slight degree of UI, which is more seldom than once a week and with small amounts. And more than half of women had stress incontinence. Our data shows that the cohort population is very similar to the source population, and the UI data also similar to previous findings from Norway [4]. As most women had slight degree of UI, they will possibly not seek clinical help. In the Cohort, UI is determined based on simple self diagnosis by women [12] and this reduces bias of the real prevalence of UI.

There are some possible limitations to the study. The age span of 40–44 years at the start is rather narrow and makes the Cohort exclusive for middle-age women. However, this was done on purpose, for concentrating the analyses for the peri- and postmenopausal decade. Although including more than 2,000 women, the statistical power may be a problem due to small sample size for some subgroups and sub-analyses.

Many cross-sectional studies have investigated continence status and associated risk factors. But there are not many cohort studies for UI, especially in middle-age women, and many previous studies are limited in duration and number of intervals [2]. Many have focused on specific subjects like pregnancy, diseases or other subgroups of women. Most cohort studies on UI in healthy middle-aged women are short and have not more than two waves [3,4,6,19-25].

Our data shows that the cohort population is very similar to the source population, and the UI data also similar to previous findings from Norway. Thus the external and internal validity of the study is expected to be good. This cohort study is therefore a potentially good tool for prospective analyses of incidence, remission and development of UI, including type and severity considerations, and associated risk factors.

## Competing interests

The authors declare that they have no competing interests.

## Authors' contributions

JD carried out the studies, analyzed and interpreted the data, and drafted the manuscript. QSA contributed as co-researcher and wrote the background and helped with the analysis of data. HS designed the study and contributed as supervisor and provided all scientific and technical supports.

## Pre-publication history

The pre-publication history for this paper can be accessed here:


